# Daytime sleepiness and the association between nocturia and depressive symptoms: A cross-sectional study

**DOI:** 10.1097/MD.0000000000049814

**Published:** 2026-07-17

**Authors:** Qiyu He, Zhimin Tan, Siyi Gu, Xian Zhang, Jinghan Yang, Xiaoqiang Li

**Affiliations:** a Department of Urology, West China Hospital, Sichuan University, Chengdu, China; b Department of Anesthesiology, West China Hospital, Sichuan University, Chengdu, China; c Department of Rehabilitation, Second Affiliated Hospital of Chongqing Medical University, Chongqing, China; d Department of Anesthesiology and Operation Center, West China Tianfu Hospital, Sichuan University, Chengdu, China.

**Keywords:** daytime sleepiness, depressive symptoms, direct and indirect associations, nocturia, sleep duration

## Abstract

Nocturia is associated with sleep disturbance and depressive symptoms, but the role of daytime sleepiness in this relationship remains unclear. This study examined the relationships among nocturia, sleep duration, daytime sleepiness, and depressive symptoms, with particular attention to the indirect associations through sleep duration and daytime sleepiness. Data from the 2015 to 2023 National Health and Nutrition Examination Survey were analyzed. Depressive symptoms were assessed using the Patient Health Questionnaire-9 and categorized as none or minimal, mild, moderate, moderately severe, or severe. Survey-weighted logistic regression was used to examine factors associated with nocturia. Interaction and direct and indirect association analyses were performed using linear regression and structural equation modeling with bootstrap confidence intervals. Among 17,731 participants, 6479 (36.5%) reported nocturia. Analyses involving daytime sleepiness included 12,634 participants. Nocturia frequency and daytime sleepiness were associated with higher Patient Health Questionnaire-9 scores, and a significant interaction was observed between them (β = 0.12, *P* < .001). The indirect association through sleep duration was not statistically significant (*P* = .083). The indirect association through daytime sleepiness was β = 0.136 (*P* < .001), representing 21.15% of the total association between nocturia and depressive symptoms. Nocturia was associated with depressive symptoms, and an indirect association through daytime sleepiness was observed. The temporal relationship among nocturia, daytime sleepiness, and depressive symptoms remains to be determined.

## 1. Introduction

Nocturia is a common urinary symptom that can substantially impair quality of life, and nocturnal polyuria is an important contributor to nocturia. Nocturnal polyuria has been reported in 31.5% of men and 38.5% of women.^[1,2]^ Repeated awakenings to void may disrupt sleep continuity and reduce slow-wave and rapid eye movement sleep.^[3,4]^ Nocturia-related sleep disturbance has been associated with daytime sleepiness, mood symptoms, impaired cognitive function, and poorer quality of life.^[5]^

Treatment of nocturia may improve sleep quality and general well-being.^[6]^ However, many patients do not regard nocturia as a medical problem, even when daytime sleepiness is present. Women may be reluctant to report urinary symptoms because of embarrassment and may attempt lifestyle changes, such as adjusting their sleep schedules, to manage these symptoms.^[7]^ Such measures may provide temporary relief but may also delay medical assessment and appropriate treatment.

An important question is whether sleep duration adequately reflects the sleep-related burden associated with nocturia. Frequent nocturnal awakenings and fragmented sleep may be more relevant than total sleep duration alone.^[3,4]^ These disturbances may reduce restorative sleep and affect emotional regulation, cognitive function, and daytime performance.^[5]^ Previous studies have linked nocturia-related sleep disruption with daytime sleepiness, mood symptoms, and reduced social functioning.^[6-9]^ Daytime sleepiness may therefore provide additional information about the functional consequences of nocturia that is not captured by sleep duration alone.

**Figure 1.**
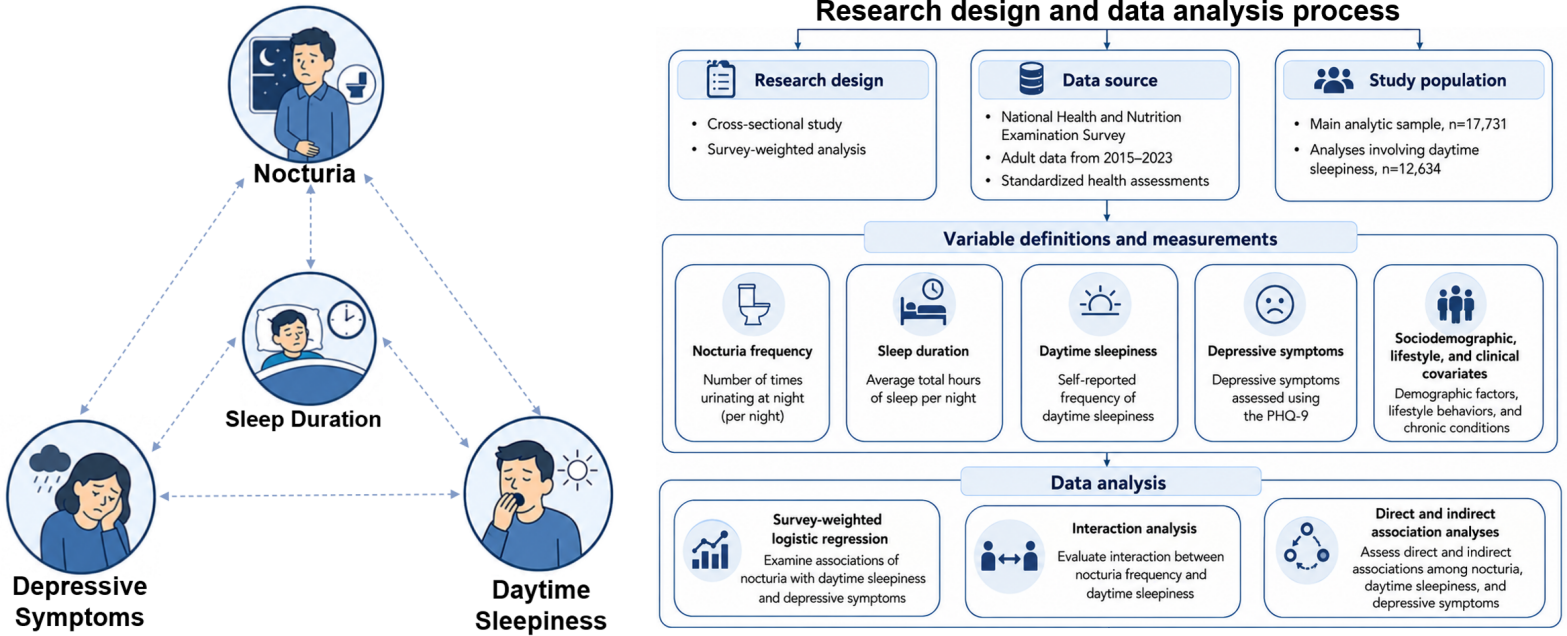
Graphical overview of the study design and examined associations. Arrows indicate the relationships evaluated in the analyses.

Accordingly, the present study examined the associations among nocturia, sleep duration, daytime sleepiness, and depressive symptoms. It further evaluated whether the indirect association through daytime sleepiness accounted for a greater proportion of the association between nocturia and depressive symptoms than the indirect association through sleep duration (Fig. 1).

## 2. Materials and methods

### 2.1. Study design

This cross-sectional study was reported in accordance with the Strengthening the Reporting of Observational Studies in Epidemiology reporting checklist. Data were obtained from the National Health and Nutrition Examination Survey (NHANES), a national survey designed to assess the health and nutritional status of the US population.^[10]^ The survey protocols were approved by the National Center for Health Statistics Research Ethics Review Board, and all participants provided written informed consent.^[11]^ The present study used publicly available, de-identified data; therefore, no additional ethical approval was required. Participants aged 20 to 80 years with available nocturia questionnaire data from the 2015 to 2016, 2017 to 2020, and 2021 to 2023 NHANES cycles were eligible. Participants with missing or invalid data required for the main analysis were excluded.

### 2.2. Measurement of nocturia

Nocturia frequency was assessed using data from the NHANES 2015 to 2023 questionnaire, which posed the question: “During the past 30 days, how many times per night did you typically get up to urinate, from the time you went to bed at night until the time you got up in the morning?” Responses were categorized on a scale from 0 to 5, where 0 indicated no episodes, 1 indicated 1 episode, 2 indicated 2 episodes, 3 indicated 3 episodes, 4 indicated 4 episodes, and 5 indicated 5 or more episodes\night. For the present analysis, clinically significant nocturia was defined as 2 or more episodes\night, a threshold associated with impaired quality of life.^[12-14]^

### 2.3. Measurement of daytime sleepiness and sleep duration

Daytime sleepiness was assessed using the NHANES question, “In the past month, how often did you feel excessively or overly sleepy during the day?” Responses were scored from 0 to 4, with 0 indicating “never,” 1 “rarely” (1 time/mo), 2 “sometimes” (2–4 times/mo), 3 “usually” (5–15 times/mo), and 4 “almost always” (16–30 times/mo). This item was available in the 2015 to 2016 and 2017 to 2020 cycles but was not collected in the 2021 to 2023 cycle; analyses involving daytime sleepiness therefore included 12,634 participants. Weekday and weekend sleep duration were calculated from the usual sleep and wake times reported for the preceding month. Sleep duration was analyzed as a continuous variable and was also categorized as short (<6 hours), 6 to 9 hours, or long (>9 hours).

### 2.4. Assessment and classification of depressive symptoms

Depressive symptoms were assessed using the Patient Health Questionnaire-9 (PHQ-9). The PHQ-9 evaluates 9 depressive symptoms experienced during the preceding 2 weeks, with each item scored from 0 to 3 and the total score ranging from 0 to 27. As a screening instrument, the PHQ-9 reflects depressive symptom severity but does not establish a clinical diagnosis. The PHQ-9 score was analyzed as a continuous variable and was also categorized as none or minimal (0–4), mild (5–9), moderate (10–14), moderately severe (15–19), or severe (20–27).^[15,16]^

### 2.5. Measurement of covariates

Potential covariates were selected based on previous studies and their availability across the included NHANES cycles. Sociodemographic variables included age, sex, body mass index (BMI), race and ethnicity, education level, and marital status. Lifestyle variables included smoking, alcohol consumption, daily sitting time, and weekly moderate- and vigorous-intensity physical activity. Clinical variables included hypertension,^[17]^ diabetes mellitus,^[18]^ chronic kidney disease (CKD),^[19]^ kidney stone history,^[20]^ and sleep-disordered breathing (SDB) symptoms.^[21]^

SDB symptoms were assessed using the question, “In the past 12 months, how often did you snort, gasp, or stop breathing while asleep?” Responses were categorized as never, rarely (1–2 nights/wk), occasionally (3–4 nights/wk), or frequently (≥5 nights/wk). This questionnaire-based measure was considered an indicator of SDB symptoms rather than a clinical diagnosis. Any urinary leakage and urgency urinary incontinence were additionally included in a sensitivity analysis. Sociodemographic characteristics, lifestyle factors, and medical history were obtained from standardized NHANES questionnaires and examination data.

### 2.6. Statistical analysis

Analyses incorporated the NHANES survey weights, strata, and primary sampling units to account for nonresponse, stratification, and oversampling. Cycle-specific weights were combined and adjusted in accordance with NHANES analytic guidance.^[22]^ Survey-weighted univariable and multivariable logistic regression models were used to examine factors associated with nocturia.

Covariates in the primary multivariable model were selected based on previous studies and clinical relevance and included the sociodemographic, lifestyle, and clinical variables described above. Depressive symptoms, sleep duration, and daytime sleepiness were excluded from the primary model because they may be involved in the association under investigation. These variables were included in a separate exploratory model. The primary and exploratory models were compared using the same complete-case sample. A further sensitivity analysis additionally adjusted for any urinary leakage and urgency urinary incontinence.

To assess possible selection bias, baseline characteristics were compared between adults included in the main analysis and those excluded because of missing or invalid data. Absolute standardized mean differences were calculated, with values >0.10 considered potentially meaningful. Multiple linear regression was used to examine the associations of nocturia frequency, daytime sleepiness, and their interaction with PHQ-9 scores. Structural equation modeling with bootstrap confidence intervals was used to estimate the direct, indirect, and total associations among nocturia, sleep duration, daytime sleepiness, and depressive symptoms.^[22,23]^ Because all variables were measured cross-sectionally, the indirect associations were not interpreted as evidence of temporal or causal pathways. Analyses were conducted using R version 4.2.3. All tests were 2-sided, and *P* < .05 was considered statistically significant.

## 3. Results

### 3.1. Demographic and clinical characteristics

**Figure 2.**
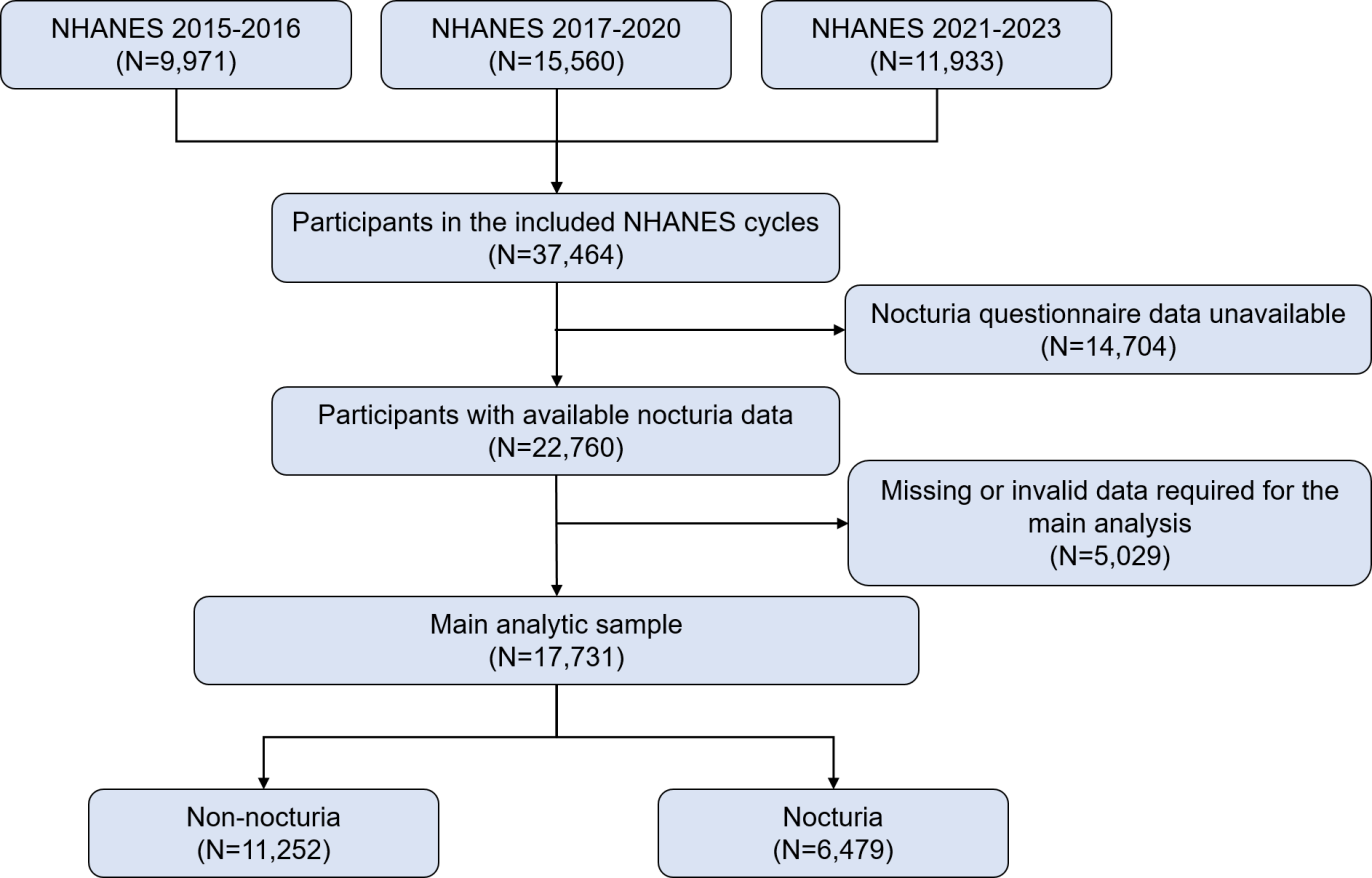
Flowchart of participant selection. Among 37,464 participants in the included NHANES cycles, 22,760 had available nocturia questionnaire data, and 17,731 were included in the main analysis. Analyses involving daytime sleepiness included 12,634 participants with available data. NHANES = National Health and Nutrition Examination Survey.

Across the 3 included NHANES cycles, 37,464 participants were identified. Nocturia questionnaire data were unavailable for 14,704 participants, leaving 22,760 participants with available nocturia data. Of these, 5029 were excluded because of missing or invalid data required for the main analysis, yielding a final analytic sample of 17,731 participants (Fig. 2). Included and excluded participants had generally similar measured characteristics, with absolute standardized mean differences ranging from 0.010 to 0.111; the largest difference was observed for college-level education (Table S1, Supplemental Digital Content 1).

Among the 17,731 participants, 6479 (36.5%) reported nocturia. Compared with participants without nocturia, those with nocturia were older (57.2 vs 47.8 years, *P* < .001), included a higher proportion of women (54.3% vs 50.9%, *P* < .001), and had a higher mean BMI (31.07 vs 29.25 kg/m^2^, *P* < .001). Non-Hispanic Black and divorced participants were more highly represented in the nocturia group. Hypertension, diabetes mellitus, CKD, and kidney stone history were also more common among participants with nocturia (Tables 1 and S2, Supplemental Digital Content 12).

**Table 1:** Demographics and clinical characteristics.

Variables	Total (N = 17,731)	Non-nocturia (N = 11,252)	Nocturia (N = 6479)	*P*-value
Individual characteristics				
Age (yr), mean (SD)	51.24 (17.5)	47.81 (17.2)	57.21 (16.3)	<.001
Age (yr), n (%)				.019
20–34	4006 (22.6)	2601 (23.1)	1405 (21.7)	
35–64	8980 (50.7)	5613 (49.9)	3367 (52.0)	
65–80	4745 (26.8)	3038 (27.0)	1707 (26.3)	
Sex, n (%)				<.001
Male	8487 (47.9)	5525 (49.1)	2962 (45.7)	
Female	9244 (52.1)	5727 (50.9)	3517 (54.3)	
BMI (kg/m^2^), mean (SD)	29.91 (7.4)	29.25 (7.0)	31.07 (7.9)	<.001
BMI (kg/m^2^), n (%)				<.001
<18.5	235 (1.3)	202 (1.8)	33 (0.5)	
18.5–24.9	4303 (24.5)	3366 (30.1)	937 (14.7)	
25–29.9	5612 (32.0)	3847 (34.4)	1765 (27.7)	
≥30	7405 (42.2)	3759 (33.6)	3646 (57.1)	
Race, n (%)				<.001
Mexican American	2091 (11.8)	1370 (12.2)	721 (11.1)	
Other Hispanic	1910 (10.8)	1192 (10.6)	718 (11.1)	
Non-Hispanic White	7583 (42.8)	4937 (43.9)	2646 (40.8)	
Non-Hispanic Black	3662 (20.7)	1998 (17.8)	1664 (25.7)	
Other race	2485 (14.0)	1755 (15.6)	730 (11.3)	
Education, n (%)				<.001
<9th grade	1236 (7.0)	638 (5.7)	598 (9.2)	
9–11th grade (includes 12th grade with no diploma)	1755 (9.9)	945 (8.4)	810 (12.5)	
High school graduate/GED or equivalent	3984 (22.5)	2352 (20.9)	1632 (25.2)	
Some college or AA degree	5639 (31.8)	3642 (32.4)	1997 (30.8)	
College graduate or above	5108 (28.8)	3670 (32.6)	1438 (22.2)	
Marital status, n (%)				<.001
Married	9736 (54.9)	6419 (57.0)	3317 (51.2)	
Divorced	3347 (18.9)	1725 (15.3)	1622 (25.0)	
Widowed	3104 (17.5)	2025 (18.0)	1079 (16.7)	
Separated	170 (1.0)	101 (0.9)	69 (1.1)	
Never married	891 (5.0)	634 (5.6)	257 (4.0)	
Living with partner	474 (2.7)	343 (3.0)	131 (2.0)	
Pregnant, n (%)	168 (4.7)	79 (0.7)	89 (1.4)	<.001
Lifestyle habits				
Smoking history, n (%)	3075 (41.2)	1895 (16.8)	1180 (18.2)	<.001
Alcohol drinking history (drinks/wk), mean (SD)	2.59 (2.2)	2.57 (2.2)	2.64 (2.3)	.090
Alcohol drinking history (drinks/wk), n (%)				<.001
Abstinent or low-risk drinking	6294 (50.0)	4221 (37.5)	2073 (32.0)	
Moderate drinking	3821 (30.4)	2558 (22.7)	1263 (19.5)	
Heavy drinking	2474 (19.7)	1615 (14.4)	859 (13.3)	
Sitting time/d (h), n (%)				<.001
<3 h/d	2646 (15.0)	1644 (14.6)	1002 (15.5)	
3–6 h/d	8678 (49.3)	5420 (48.2)	3258 (50.3)	
6–9 h/d	3208 (18.2)	2049 (18.2)	1159 (17.9)	
>9 h/d	3078 (17.5)	2068 (18.4)	1010 (15.6)	
Weekly high-intensity exercise time, mean (SD)	260.80 (348.3)	252.97 (323.9)	277.62 (395.5)	.005
Weekly high-intensity exercise time, n (%)				.001
Inactive	141 (2.0)	81 (1.6)	60 (2.6)	
Insufficiently active	1376 (19.1)	899 (18.3)	477 (20.9)	
Sufficiently active	1813 (25.2)	1263 (25.7)	550 (24.2)	
Highly active	3869 (53.7)	2679 (54.4)	1190 (52.3)	
Weekly mid-intensity exercise time, mean (SD)	196.90 (284.6)	190.80 (258.4)	210.50 (334.9)	.005
Weekly mid-intensity exercise time, n (%)				.003
Inactive	295 (3.9)	186 (3.6)	109 (4.6)	
Insufficiently active	4065 (53.7)	2792 (53.6)	1273 (53.7)	
Sufficiently active	2049 (27.0)	1458 (28.0)	591 (24.9)	
Highly active	1167 (15.4)	771 (14.8)	396 (16.7)	
Underlying diseases				
Hypertension, n (%)	6665 (37.6)	3385 (30.1)	3280 (50.6)	<.001
Diabetes mellitus, n (%)	2611 (14.7)	1168 (10.4)	1443 (22.3)	<.001
CKD, n (%)	700 (4.0)	308 (2.7)	392 (6.1)	<.001
Kidney stone history, n (%)	1274 (7.2)	778 (6.9)	496 (7.7)	<.001
Severity of depressive symptoms				
Depression score, mean (SD)	3.50 (4.4)	2.95 (4.0)	4.37 (4.9)	<.001
Depression level, n (%)				<.001
No depression	12,833 (72.4)	8724 (77.5)	4109 (63.4)	
Mild depression	3161 (17.8)	1678 (14.9)	1483 (22.9)	
Moderate depression	1117 (6.3)	568 (5.0)	549 (8.5)	
Moderately severe depression	436 (2.5)	199 (1.8)	237 (3.7)	
Severe depression	184 (1.0)	83 (0.7)	101 (1.6)	
Sleep pattern and daytime sleepiness				
Sleep-disordered breathing symptoms, n (%)				<.001
Never	8878 (74.5)	5755 (75.6)	3123 (72.5)	
1–2 nights/wk	1598 (13.4)	1009 (13.3)	589 (13.7)	
3–4 nights/wk	776 (6.5)	475 (6.2)	301 (7.0)	
≥5 nights/wk	669 (5.6)	376 (4.9)	293 (6.8)	
Weekday sleep hours, mean (SD)	7.60 (1.6)	7.55 (1.5)	7.75 (1.8)	<.001
Weekday sleep hours, n (%)				<.001
<6	1718 (9.7)	996 (8.9)	722 (11.1)	
6–9	14,048 (79.2)	9282 (82.5)	4766 (73.6)	
>9	1965 (11.1)	974 (8.7)	991 (15.3)	
Weekend sleep hours, mean (SD)	8.20 (1.7)	8.22 (1.6)	8.22 (1.9)	.875
Weekend sleep hours, n (%)				<.001
<6	847 (6.6)	440 (5.5)	407 (8.4)	
6–9	9213 (71.8)	5945 (74.1)	3268 (67.8)	
>9	2778 (21.6)	1633 (20.4)	1145 (23.8)	
Daytime sleepiness score, mean (SD)	1.75 (1.2)	1.68 (1.1)	1.88 (1.2)	<.001

Data are present in n (%) and mean (SD).

AA = associate of arts, BMI = body mass index, CKD = chronic kidney disease, GED = general educational development, SD = standard deviation.

Participants with nocturia had higher proportions of both short (<6 hours) and long (>9 hours) weekday sleep and more frequent SDB symptoms. Among participants with available daytime sleepiness data, the mean daytime sleepiness score was higher in the nocturia group than in the non-nocturia group (1.88 vs 1.68, *P* < .001). The mean PHQ-9 score was also higher among participants with nocturia (4.37 vs 2.95, *P* < .001). Daytime sleepiness and PHQ-9 scores increased with nocturia frequency (Tables 1 and S2, Supplemental Digital Content 12).

### 3.2. Univariable and multivariable analyses of factors associated with nocturia

Univariable analysis showed that nocturia was associated with several demographic, lifestyle, clinical, sleep-related, and psychological variables, including age of 35 to 64 years, female sex, higher BMI, non-Hispanic Black race, lower education level, divorced status, smoking, heavy alcohol consumption, hypertension, diabetes mellitus, CKD, depressive symptoms, sleep duration, and daytime sleepiness (Table 2).

**Table 2:** Univariable logistic regression analysis of factors associated with nocturia.

Variables	β	Standard error	*t*-value	*P*-value	OR (95% CI)
Individual characteristics					
Age (yr)					
20–34	Reference	Reference	Reference	Reference	Reference
35–64	0.10	0.04	2.64	.008	1.11 (1.03, 1.20)
65–80	0.04	0.04	0.88	.380	1.04 (0.95, 1.14)
Female	0.13	0.04	2.95	.005	1.14 (1.04, 1.24)
BMI (kg/m^2^)					
<18.5	Reference	Reference	Reference	Reference	Reference
18.5–24.9	0.83	0.25	3.31	.002	2.30 (1.39, 3.81)
25–29.9	1.30	0.25	5.14	<.001	3.68 (2.21, 6.11)
≥30	2.05	0.25	8.34	<.001	7.75 (4.74, 12.68)
Race					
Mexican American	Reference	Reference	Reference	Reference	Reference
Other Hispanic	0.22	0.09	2.40	.020	1.24 (1.04, 1.49)
Non-Hispanic White	0.07	0.07	0.94	.354	1.07 (0.92, 1.24)
Non-Hispanic Black	0.53	0.09	5.98	<.001	1.70 (1.42, 2.02)
Other race	−0.22	0.09	−2.46	.017	0.81 (0.68, 0.96)
Education					
<9th grade	Reference	Reference	Reference	Reference	Reference
9–11th grade (Includes 12th grade with no diploma)	−0.11	0.09	−1.13	.265	0.90 (0.75, 1.09)
High school graduate/GED or equivalent	−0.26	0.09	−2.93	.005	0.77 (0.64, 0.92)
Some college or AA degree	−0.55	0.08	−6.85	<.001	0.58 (0.49, 0.68)
College graduate or above	−0.81	0.09	−8.80	<.001	0.45 (0.37, 0.54)
Marital status					
Married	Reference	Reference	Reference	Reference	Reference
Divorced	0.63	0.06	10.21	<.001	1.88 (1.66, 2.13)
Widowed	0.17	0.07	2.35	.023	1.19 (1.03, 1.37)
Separated	0.07	0.20	0.36	.718	1.08 (0.72, 1.62)
Never married	−0.26	0.09	−2.74	.008	0.77 (0.64, 0.93)
Living with partner	−0.36	0.12	−3.05	.004	0.70 (0.55, 0.89)
Lifestyle habits					
Smoking history	0.17	0.07	2.36	.022	1.18 (1.02, 1.36)
Alcohol drinking history (drinks/wk)					
Abstinent or low-risk drinking	Reference	Reference	Reference	Reference	Reference
Moderate drinking	0.02	0.05	0.29	.776	1.02 (0.91, 1.13)
Heavy drinking	0.21	0.07	3.15	.003	1.24 (1.08, 1.42)
Sitting time/d (h)					
<3 h/d	Reference	Reference	Reference	Reference	Reference
3–6 h/d	0.02	0.07	0.34	.734	1.02 (0.89, 1.18)
6–9 h/d	−0.01	0.08	−0.15	.881	0.99 (0.85, 1.15)
>9 h/d	−0.14	0.08	−1.73	.089	0.87 (0.74, 1.02)
Underlying diseases					
Hypertension	0.87	0.03	26.95	<.001	2.39 (2.24, 2.54)
Diabetes mellitus	0.61	0.03	18.74	<.001	1.84 (1.73, 1.97)
CKD	0.83	0.08	10.66	<.001	2.29 (1.96, 2.67)
Kidney stone history	0.20	0.09	2.27	.023	1.22 (1.03, 1.44)
Severity of depressive symptoms					
No depression	Reference	Reference	Reference	Reference	Reference
Mild depression	0.70	0.05	12.76	<.001	2.01 (1.80, 2.24)
Moderate depression	0.79	0.10	8.23	<.001	2.20 (1.81, 2.66)
Moderately severe depression	0.93	0.13	6.95	<.001	2.55 (1.94, 3.34)
Severe depression	1.07	0.21	5.05	<.001	2.91 (1.90, 4.46)
Sleep pattern and daytime sleepiness					
Sleep-disordered breathing symptoms					
Never	Reference	Reference	Reference	Reference	Reference
1–2 nights/wk	0.07	0.06	1.30	.20	1.08 (0.96, 1.20)
3–4 nights/wk	0.16	0.08	2.01	.04	1.17 (1.00, 1.36)
≥5 nights/wk	0.36	0.08	4.47	<.001	1.44 (1.22, 1.68)
Weekday sleep hours					
<6	Reference	Reference	Reference	Reference	Reference
6–9	−0.34	0.05	−6.6	<.001	0.71 (0.64, 0.78)
>9	0.23	0.10	2.28	.023	1.26 (1.03, 1.54)
Daytime sleepiness score					
0	Reference	Reference	Reference	Reference	Reference
1	−0.11	0.08	−1.44	.157	0.89 (0.76, 1.05)
2	0.08	0.08	1.03	.308	1.09 (0.92, 1.28)
3	0.22	0.07	3.20	.002	1.25 (1.09, 1.44)
4	0.58	0.12	4.83	<.001	1.78 (1.40, 2.27)

AA = associate of arts, BMI = body mass index, CI = confidence interval, CKD = chronic kidney disease, GED = general educational development, OR = odds ratio.

In separate multivariable models, higher BMI, female sex, non-Hispanic Black race, lower education level, and divorced or widowed status were associated with higher odds of nocturia (Table S3, Supplemental Digital Content 2). Moderate-intensity physical activity was inversely associated with nocturia, whereas smoking and alcohol consumption were not associated with nocturia (Table S4, Supplemental Digital Content 3). Hypertension, diabetes mellitus, and CKD were associated with higher odds of nocturia, whereas kidney stone history was not (Table S5, Supplemental Digital Content 4). Depressive symptom severity, sleep duration, and daytime sleepiness were examined in a separate model (Table S6, Supplemental Digital Content 5).

**Figure 3.**
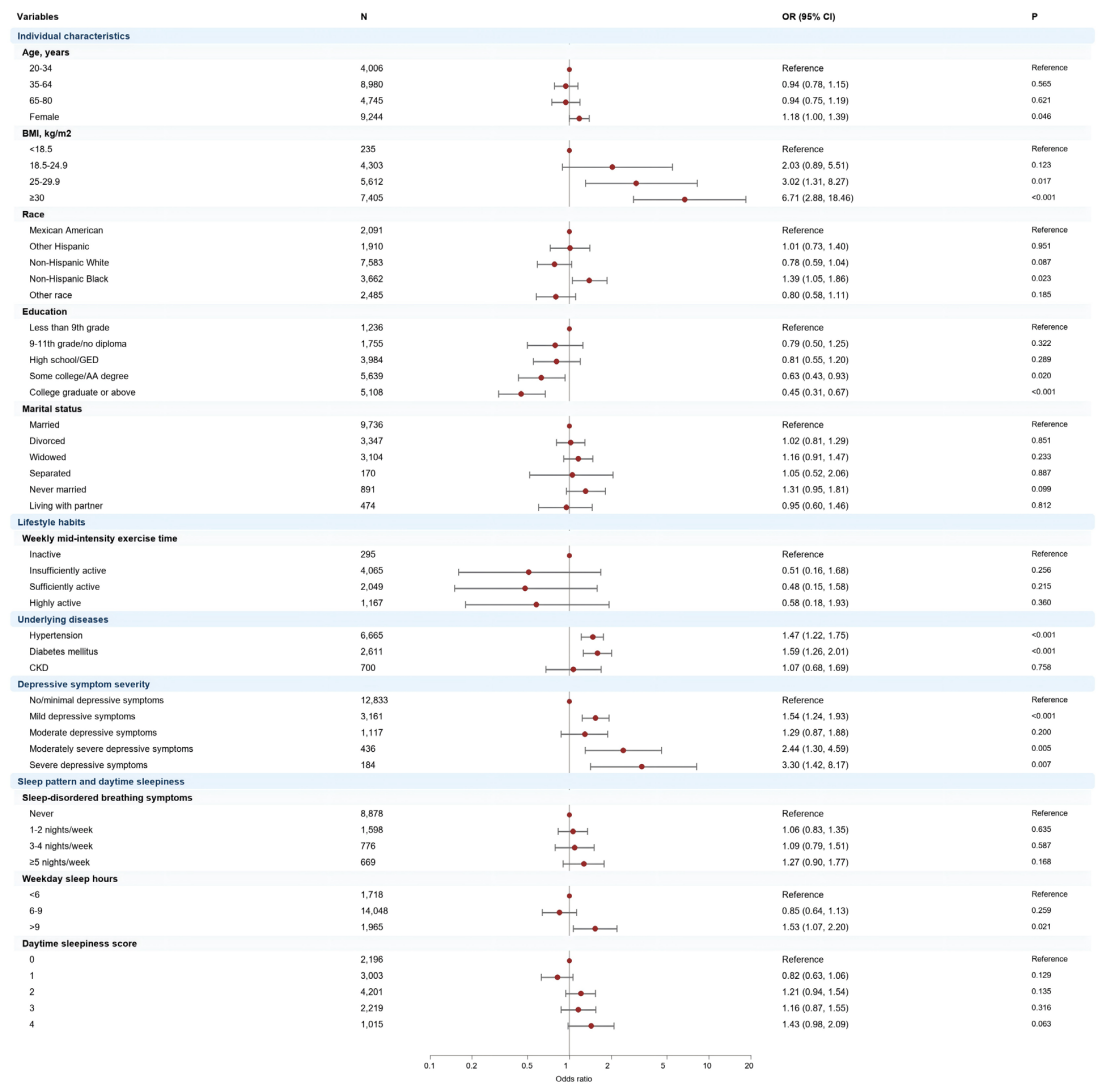
Forest plot of the exploratory multivariable logistic regression model for nocturia. The model included depressive symptoms, sleep duration, and daytime sleepiness in addition to demographic, lifestyle, and clinical variables. AA = associate of arts, CKD = chronic kidney disease, GED = general educational development, OR = odds ratio, Ref = reference.

Depressive symptoms, sleep duration, and daytime sleepiness were excluded from the primary adjustment model to reduce possible overadjustment. When these variables were added to an exploratory model using the same complete-case sample, the estimates for female sex, non-Hispanic Black race, hypertension, and diabetes mellitus remained similar (Table S7, Supplemental Digital Content 6). In the exploratory model, BMI ≥ 30 kg/m^2^, female sex, non-Hispanic Black race, hypertension, diabetes mellitus, moderate and moderately severe depressive symptoms, and weekday sleep duration >9 hours were associated with nocturia (Table 3 and Fig. 3). Additional adjustment for any urinary leakage and urgency urinary incontinence produced similar estimates (Table S8, Supplemental Digital Content 7).

**Table 3:** Exploratory multivariable logistic regression analysis of factors associated with nocturia (model 5).

Variables	β	Standard error	*t*-value	*P*-value	OR (95% CI)
Individual characteristics					
Age (yr)					
20–34	Reference	Reference	Reference	Reference	Reference
35–64	−0.06	0.10	−0.58	.565	0.94 (0.78, 1.15)
65–80	−0.06	0.12	−0.50	.621	0.94 (0.75, 1.19)
Female	0.16	0.08	1.99	.046	1.18 (1.00, 1.39)
BMI (kg/m^2^)					
<18.5	Reference	Reference	Reference	Reference	Reference
18.5–24.9	0.71	0.46	1.54	.123	2.03 (0.89, 5.51)
25–29.9	1.11	0.46	2.39	.017	3.02 (1.31, 8.27)
≥30	1.90	0.47	4.08	<.001	6.71 (2.88, 18.46)
Race					
Mexican American	Reference	Reference	Reference	Reference	Reference
Other Hispanic	0.01	0.17	0.06	.951	1.01 (0.73, 1.40)
Non-Hispanic White	−0.24	0.14	−1.71	.087	0.78 (0.59, 1.04)
Non-Hispanic Black	0.33	0.15	2.27	.023	1.39 (1.05, 1.86)
Other race	−0.22	0.16	−1.32	.185	0.80 (0.58, 1.11)
Education					
<9th grade	Reference	Reference	Reference	Reference	Reference
9–11th grade (Includes 12th grade with no diploma)	−0.23	0.23	−0.99	.322	0.79 (0.50, 1.25)
High school graduate/GED or equivalent	−0.21	0.20	−1.06	.289	0.81 (0.55, 1.20)
Some college or AA degree	−0.46	0.20	−2.34	.020	0.63 (0.43, 0.93)
College graduate or above	−0.79	0.20	−3.98	<.001	0.45 (0.31, 0.67)
Marital status					
Married	Reference	Reference	Reference	Reference	Reference
Divorced	0.02	0.12	0.19	.851	1.02 (0.81, 1.29)
Widowed	0.15	0.12	1.19	.233	1.16 (0.91, 1.47)
Separated	0.05	0.35	0.14	.887	1.05 (0.52, 2.06)
Never married	0.27	0.16	1.65	.099	1.31 (0.95, 1.81)
Living with partner	−0.05	0.22	−0.24	.812	0.95 (0.60, 1.46)
Lifestyle habits					
Weekly mid-intensity exercise time					
Inactive	Reference	Reference	Reference	Reference	Reference
Insufficiently active	−0.67	0.59	−1.13	.256	0.51 (0.16, 1.68)
Sufficiently active	−0.74	0.60	−1.24	.215	0.48 (0.15, 1.58)
Highly active	−0.55	0.60	−0.92	.360	0.58 (0.18, 1.93)
Underlying diseases					
Hypertension	0.38	0.09	4.16	<.001	1.47 (1.22, 1.75)
Diabetes mellitus	0.46	0.12	3.87	<.001	1.59 (1.26, 2.01)
CKD	0.07	0.23	0.31	.758	1.07 (0.68, 1.69)
Severity of depressive symptoms					
No depression	Reference	Reference	Reference	Reference	Reference
Mild depression	0.43	0.11	3.83	<.001	1.54 (1.24, 1.93)
Moderate depression	0.25	0.20	1.28	.200	1.29 (0.87, 1.88)
Moderately severe depression	0.89	0.32	2.79	.005	2.44 (1.30, 4.59)
Severe depression	1.20	0.44	2.71	.007	3.30 (1.42, 8.17)
Sleep Pattern and daytime sleepiness					
Sleep-disordered breathing symptoms					
Never	Reference	Reference	Reference	Reference	Reference
1–2 nights/wk	0.06	0.12	0.48	.635	1.06 (0.83, 1.35)
3–4 nights/wk	0.09	0.17	0.54	.587	1.09 (0.79, 1.51)
≥5 nights/wk	0.24	0.17	1.38	.168	1.27 (0.90, 1.77)
Weekday sleep hours					
<6	Reference	Reference	Reference	Reference	Reference
6–9	−0.16	0.14	−1.13	.259	0.85 (0.64, 1.13)
>9	0.43	0.19	2.30	.021	1.53 (1.07, 2.20)
Daytime sleepiness score					
0	Reference	Reference	Reference	Reference	Reference
1	−0.20	0.13	−1.52	.129	0.82 (0.63, 1.06)
2	0.19	0.13	1.50	.135	1.21 (0.94, 1.54)
3	0.15	0.15	1.00	.316	1.16 (0.87, 1.55)
4	0.36	0.19	1.86	.063	1.43 (0.98, 2.09)

Model 5 included individual characteristics, lifestyle factors, clinical conditions, depressive symptoms, sleep duration, and daytime sleepiness.

The latter 3 variables were excluded from the primary adjustment model to reduce possible overadjustment.

AA = associate of arts, BMI = body mass index, CI = confidence interval, CKD = chronic kidney disease, GED = general educational development, OR = odds ratio.

### 3.3. Interaction between nocturia frequency and daytime sleepiness in relation to depressive symptom severity

**Figure 4.**
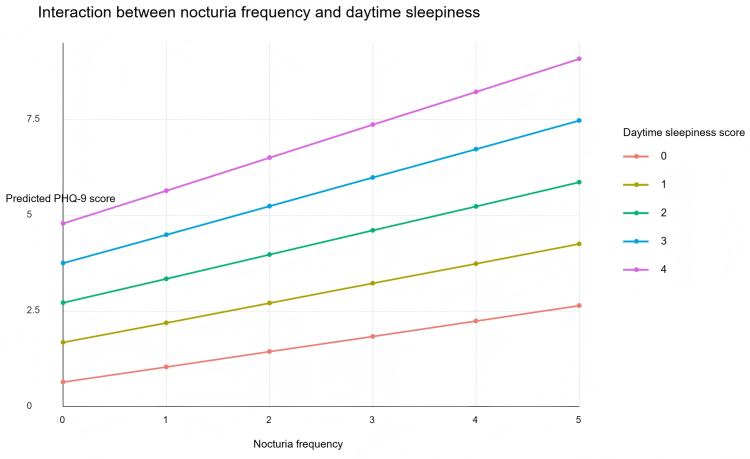
Interaction between nocturia frequency and PHQ-9 score. PHQ-9 = Patient Health Questionnaire-9.

In multiple linear regression analysis, nocturia frequency (β = 0.40, *P* < .001) and daytime sleepiness score (β = 1.04, *P* < .001) were associated with higher PHQ-9 scores. An interaction was observed between nocturia frequency and daytime sleepiness score (β = 0.12, *P* < .001), indicating that the association between nocturia frequency and PHQ-9 scores varied according to the level of daytime sleepiness (Table S9, Supplemental Digital Content 8). The association between nocturia frequency and PHQ-9 scores was weaker at lower daytime sleepiness scores and became stronger as daytime sleepiness scores increased (Fig. 4).

### 3.4. Direct and indirect associations among nocturia, sleep duration, daytime sleepiness, and depressive symptoms

**Figure 5.**
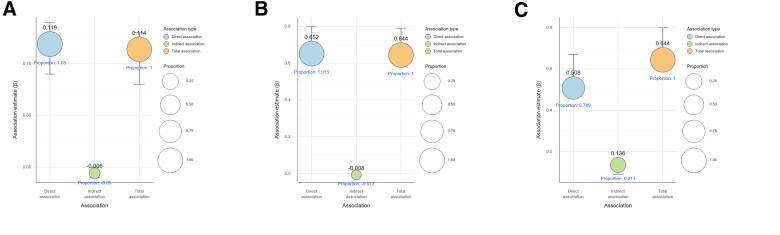
Direct and indirect association estimates with 95% confidence intervals. (A) Associations among nocturia, sleep duration, and daytime sleepiness. (B) Associations among nocturia, sleep duration, and depressive symptoms. (C) Associations among nocturia, daytime sleepiness, and depressive symptoms. Estimates were derived from cross-sectional data.

Sleep duration and daytime sleepiness were examined as potential intermediate variables in the association between nocturia and depressive symptoms. The indirect association through sleep duration was not statistically significant (β = −0.008, *P* = .083; Fig. 5A, B; Tables S10 and S11, Supplemental Digital Content 9).

**Figure 6.**
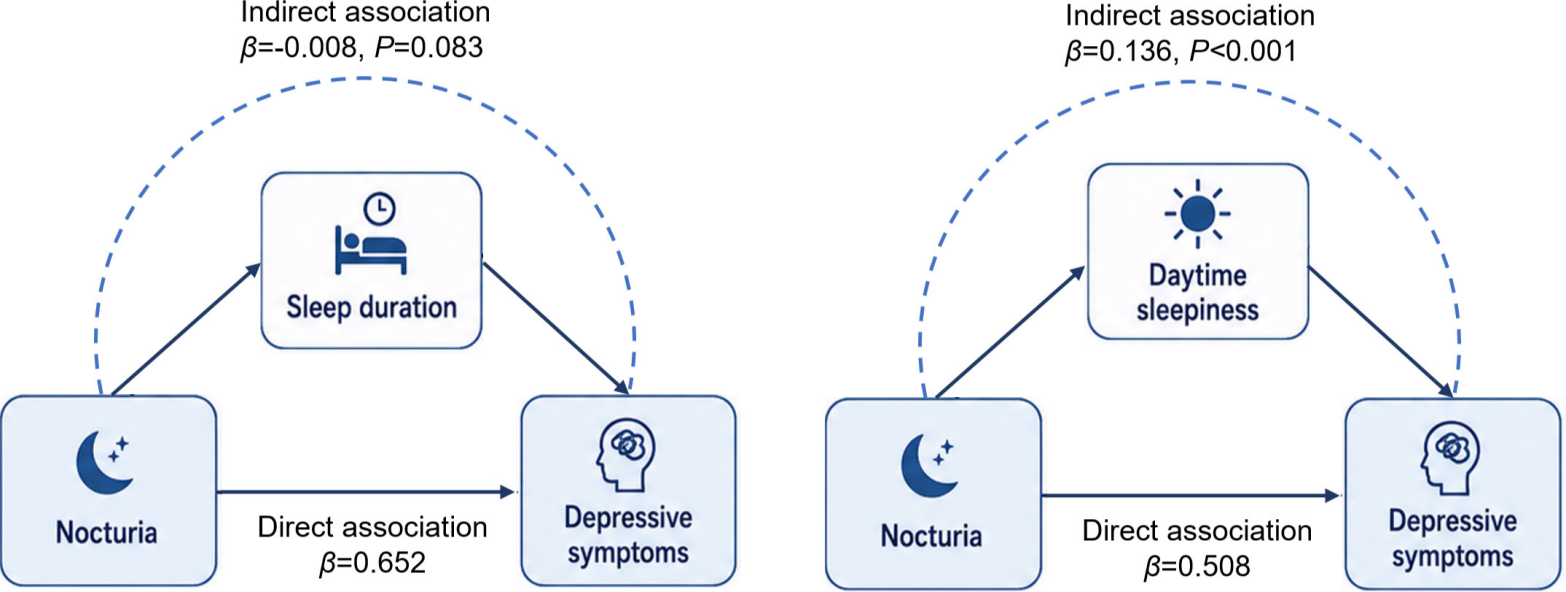
Direct and indirect associations of nocturia with depressive symptoms through sleep duration and daytime sleepiness. (A) Sleep duration. (B) Daytime sleepiness. β, regression coefficient. Estimates were derived from cross-sectional data.

The indirect association through daytime sleepiness was β = 0.136 (*P* < .001), while the direct association between nocturia and depressive symptoms was β = 0.508. The indirect association accounted for approximately 21.15% of the total association (Figs. 5C and 6; Table S12, Supplemental Digital Content 10).

## 4. Discussion

### 4.1. Key findings

Nocturia was associated with female sex, non-Hispanic Black race, higher BMI, lower education, hypertension, diabetes mellitus, depressive symptoms, and long weekday sleep duration, whereas moderate-intensity physical activity was associated with lower odds of nocturia. Nocturia frequency was associated with higher PHQ-9 scores, and this association was stronger among participants with greater daytime sleepiness. The indirect association through daytime sleepiness accounted for 21.15% of the total association between nocturia and depressive symptoms, whereas the indirect association through sleep duration was not statistically significant. Sensitivity analyses excluding potential mediators and adjusting for urinary incontinence variables yielded similar estimates.

### 4.2. Current research progress

Previous studies suggest a bidirectional relationship between nocturia and depression. The Nagahama cohort study in Japan found that higher nocturia frequency was associated with an increased likelihood of subsequent depressive symptoms, with a stronger predictive effect than the reverse pathway.^[24]^ The HEIJO-KYO cohort also confirmed that nocturia was strongly linked to the onset of new depression, with sleep disturbances potentially amplifying this effect.^[25]^ While longitudinal studies support the temporal link between nocturia and mental health, the underlying mechanisms remain unclear.

After adjustment for demographic, lifestyle, and clinical factors, nocturia frequency showed a graded association with depressive symptom severity. The indirect association through daytime sleepiness was greater than that through sleep duration, suggesting that daytime functional impairment may be more closely related to depressive symptoms than sleep duration alone. Previous research has also shown that nocturia is associated with poor sleep quality independent of age and sex (OR = 6.1, 95% CI: 1.8–25.4).^[26]^ The interaction analysis further showed that the association between nocturia frequency and PHQ-9 scores was stronger among participants with greater daytime sleepiness. These findings support consideration of daytime sleepiness when assessing the psychological burden associated with nocturia.

### 4.3. Potential mechanisms

Daytime functional impairment associated with nocturia may contribute to poorer emotional health. Repeated nocturnal awakenings may disrupt slow-wave and rapid eye movement sleep and alter sympathetic nervous system and hypothalamic-pituitary-adrenal axis activity. The resulting changes in cortisol secretion and neurotransmitter signaling may affect mood regulation and cognitive function.^[8,27,28]^

Nocturia and depressive symptoms may also share neurobiological features. Serotonergic signaling is involved in both mood regulation and lower urinary tract function.^[29]^ Circadian rhythm disturbances may alter vasopressin secretion and have also been associated with mood instability.^[8,30,31]^ In the present study, sleep duration showed a weaker indirect association than daytime sleepiness, suggesting that total sleep time alone may not fully capture the sleep-related burden associated with nocturia.

Inflammation may also serve as a link between nocturia and depression. Chronic low-grade inflammation in depression, marked by interleukin-6 and tumor necrosis factor-α, disrupts neuroendocrine and neuroplasticity processes, affecting mood stability.^[32,33]^ Nocturia, associated with inflammatory changes in some urological conditions, may similarly activate inflammation and influence mood. Factors such as female sex, obesity, hypertension, and diabetes, which are associated with nocturia, are also linked to depression, suggesting a potential comorbidity effect. In particular, women may be more vulnerable to a cycle of nocturia, sleep disruption, daytime sleepiness, and mood instability.

### 4.4. Clinical implications

These findings support the assessment of daytime sleepiness and depressive symptoms in patients presenting with nocturia. The co-occurrence of these symptoms may indicate a greater overall symptom burden and may help identify patients who could benefit from coordinated urological, sleep, and psychological evaluation. Whether reducing daytime sleepiness improves depressive symptoms remains to be determined.

A multidisciplinary approach may be appropriate for patients with nocturia and depressive symptoms. Clinical assessment should include nocturia frequency and severity, fluid intake and timing, lower urinary tract symptoms, sleep quality, medication use, mood symptoms, and relevant comorbidities, with management directed toward the contributing factors identified.^[3,14]^ Behavioral measures, including adjustment of evening fluid intake and other lifestyle factors, may be combined with treatment for overactive bladder, bladder outlet obstruction, or nocturnal polyuria when indicated.^[14]^ SDB deserves particular attention because obstructive sleep apnea is frequently associated with nocturia, and continuous positive airway pressure may reduce nocturnal voiding in some patients.^[21]^ Hypertension and diabetes mellitus should also be assessed because both are associated with nocturia and may increase the overall clinical burden.^[17,18]^ Further mental health assessment may be appropriate when depressive symptoms are prominent. In older adults, multimorbidity, polypharmacy, altered sleep, and mobility limitations may complicate symptom assessment, and management should therefore be individualized.

Nocturia involves overlapping urological, sleep, and psychological concerns. Daytime functioning should be considered alongside urinary symptoms and sleep duration during clinical assessment. Longitudinal and interventional studies are needed to determine whether addressing nocturia-related sleep disturbance and daytime sleepiness improves depressive symptoms and other patient-reported outcomes.

### 4.5. Limitations

The cross-sectional design does not establish temporal sequence, and the indirect association through daytime sleepiness should therefore be regarded as hypothesis-generating. Sleep-related measures were questionnaire-based and did not capture sleep fragmentation or other dimensions of sleep quality, and daytime sleepiness was available only in the earlier NHANES cycles. The large analytic sample, the broadly similar characteristics of included and excluded participants, and the stability of the estimates after additional adjustment for urinary leakage and urgency urinary incontinence lend support to the findings, although unmeasured urological and treatment-related factors may still have influenced the results.

## 5. Conclusions

In this cross-sectional NHANES analysis, nocturia was associated with greater daytime sleepiness and more severe depressive symptoms. Daytime sleepiness, but not sleep duration, accounted for part of this association, suggesting that daytime symptoms may better reflect the sleep-related burden of nocturia.

## Acknowledgments

The authors thank the participants and staff of the National Health and Nutrition Examination Survey and the National Center for Health Statistics for making the data publicly available.

## Author contributions

**Conceptualization:** Qiyu He, Zhimin Tan.

**Data curation:** Qiyu He, Zhimin Tan.

**Investigation:** Siyi Gu, Jinghan Yang.

**Methodology:** Siyi Gu, Xian Zhang, Jinghan Yang.

**Project administration:** Xiaoqiang Li.

**Supervision:** Xiaoqiang Li.

**Validation:** Xian Zhang.

**Writing – original draft:** Qiyu He, Zhimin Tan.

**Writing – review & editing:** Xiaoqiang Li.
























