# Supplementary material for: Daytime sleepiness and the association between nocturia and depressive symptoms: A cross-sectional study
Source: Medicine (Baltimore). 2026 Jul 17;105(29):e49814. doi: 10.1097/MD.0000000000049814 (PMC13384633; doi:10.1097/MD.0000000000049814)
Supplement: Supplementary file 1 [file medi-105-e49814-s001.docx]

**Table S1** Comparison of baseline characteristics between included and excluded adults.

| **Characteristic** | **Included adults (N=17,731)** | **Excluded adults (N=5,029)** | **Absolute SMD** |
| --- | --- | --- | --- |
| Age, years | 51.24 (17.45) | 52.82 (18.59) | 0.087 |
| Female sex | 9,244 (52.1%) | 2,802 (55.7%) | 0.072 |
| Non-Hispanic Black race | 3,662 (20.7%) | 990 (19.7%) | 0.024 |
| College graduate or above | 5,108 (28.8%) | 1,196 (24.0%) | 0.111 |
| Married | 9,736 (54.9%) | 2,565 (51.3%) | 0.073 |
| BMI, kg/m2 | 29.91 (7.35) | 29.26 (7.51) | 0.088 |
| Hypertension | 6,665 (37.6%) | 1,912 (38.1%) | 0.011 |
| Diabetes mellitus | 2,611 (14.7%) | 723 (14.4%) | 0.01 |

Values are mean (SD) or n (%). SMD, standardized mean difference. Available-case denominators vary because baseline covariates were also missing among some excluded adults.
