# Supplementary material for: Daytime sleepiness and the association between nocturia and depressive symptoms: A cross-sectional study
Source: Medicine (Baltimore). 2026 Jul 17;105(29):e49814. doi: 10.1097/MD.0000000000049814 (PMC13384633; doi:10.1097/MD.0000000000049814)
Supplement: Supplementary file 2 [file medi-105-e49814-s002.docx]

**Table S3** Multivariable logistic regression analysis of factors associated with nocturia (Model 1).

| **Individual characteristics** | **β** | **Standard Error** | ***t*-value** | **P-value** | **OR (95% CI)** |
| --- | --- | --- | --- | --- | --- |
| Age, years |  |  |  |  |  |
| 20-34 | Reference | Reference | Reference | Reference | Reference |
| 35-64 | 0.08 | 0.04 | 2.07 | 0.038 | 1.09 (1.00, 1.18) |
| 65-80 | 0.03 | 0.05 | 0.66 | 0.507 | 1.03 (0.94, 1.13) |
| Female | 0.15 | 0.03 | 4.49 | <0.001 | 1.16 (1.09, 1.24) |
| BMI, kg/m^2^ |  |  |  |  |  |
| <18.5 | Reference | Reference | Reference | Reference | Reference |
| 18.5-24.9 | 0.73 | 0.19 | 3.78 | <0.001 | 2.08 (1.44, 3.08) |
| 25-29.9 | 1.24 | 0.19 | 6.40 | <0.001 | 3.46 (2.40, 5.14) |
| ≥30 | 1.98 | 0.19 | 10.20 | <0.001 | 7.22 (5.01, 10.73) |
| Race |  |  |  |  |  |
| Mexican American | Reference | Reference | Reference | Reference | Reference |
| Other Hispanic | 0.13 | 0.07 | 1.82 | 0.069 | 1.14 (0.99, 1.30) |
| Non-Hispanic White | 0.03 | 0.06 | 0.48 | 0.628 | 1.03 (0.92, 1.16) |
| Non-Hispanic Black | 0.50 | 0.06 | 7.90 | <0.001 | 1.65 (1.46, 1.87) |
| Other race | 0.02 | 0.07 | 0.26 | 0.797 | 1.02 (0.89, 1.17) |
| Education |  |  |  |  |  |
| Less than 9th grade | Reference | Reference | Reference | Reference | Reference |
| 9-11th grade (Includes 12th grade with no diploma) | -0.04 | 0.08 | -0.52 | 0.606 | 0.96 (0.82, 1.12) |
| High school graduate/GED or equivalent | -0.27 | 0.07 | -3.66 | <0.001 | 0.77 (0.66, 0.88) |
| Some college or AA degree | -0.48 | 0.07 | -6.66 | <0.001 | 0.62 (0.54, 0.71) |
| College graduate or above | -0.78 | 0.07 | -10.57 | <0.001 | 0.46 (0.40, 0.53) |
| Marital status |  |  |  |  |  |
| Married | Reference | Reference | Reference | Reference | Reference |
| Divorced | 0.26 | 0.04 | 5.92 | <0.001 | 1.30 (1.19, 1.41) |
| Widowed | 0.26 | 0.05 | 5.45 | <0.001 | 1.30 (1.18, 1.43) |
| Separated | 0.03 | 0.17 | 0.18 | 0.855 | 1.03 (0.74, 1.42) |
| Never married | 0.07 | 0.08 | 0.83 | 0.408 | 1.07 (0.91, 1.26) |
| Living with partner | -0.04 | 0.11 | -0.38 | 0.707 | 0.96 (0.77, 1.19) |

Model 1: Including individual characteristics (age, sex, BMI, race, education, and marital status).

AA, associate of arts; BMI, body mass index; CI, confidence interval; GED, general educational development; OR, odds ratio.
