# Supplementary material for: Daytime sleepiness and the association between nocturia and depressive symptoms: A cross-sectional study
Source: Medicine (Baltimore). 2026 Jul 17;105(29):e49814. doi: 10.1097/MD.0000000000049814 (PMC13384633; doi:10.1097/MD.0000000000049814)
Supplement: Supplementary file 3 [file medi-105-e49814-s003.docx]

**Table S4** Multivariable logistic regression analysis of factors associated with nocturia (Model 2).

| **Lifestyle habits** | **β** | **Standard Error** | ***t*-value** | **P-value** | **OR (95% CI)** |
| --- | --- | --- | --- | --- | --- |
| Smoking history | <0.01 | 0.14 | 0.02 | 0.981 | 1.00 (0.77, 1.31) |
| Alcohol drinking history (drinks/week) |  |  |  |  |  |
| Abstinent or low-risk drinking | Reference | Reference | Reference | Reference | Reference |
| Moderate drinking | 0.01 | 0.15 | 0.09 | 0.932 | 1.01 (0.76, 1.35) |
| Heavy drinking | -0.26 | 0.17 | -1.55 | 0.120 | 0.77 (0.56, 1.07) |
| Sitting time/day (h) |  |  |  |  |  |
| <3 hours/day | Reference | Reference | Reference | Reference | Reference |
| 3-6 hours/day | -0.05 | 0.18 | -0.28 | 0.777 | 0.95 (0.68, 1.35) |
| 6-9 hours/day | -0.16 | 0.22 | -0.73 | 0.463 | 0.85 (0.55, 1.31) |
| >9 hours/day | -0.12 | 0.22 | -0.55 | 0.586 | 0.89 (0.57, 1.37) |
| Weekly high-intensity exercise time |  |  |  |  |  |
| Inactive | Reference | Reference | Reference | Reference | Reference |
| Insufficiently active | -0.51 | 0.64 | -0.79 | 0.428 | 0.60 (0.17, 2.22) |
| Sufficiently active | -0.92 | 0.64 | -1.43 | 0.153 | 0.40 (0.11, 1.48) |
| Highly active | -0.30 | 0.63 | -0.47 | 0.635 | 0.74 (0.21, 2.70) |
| Weekly mid-intensity exercise time |  |  |  |  |  |
| Inactive | Reference | Reference | Reference | Reference | Reference |
| Insufficiently active | -0.81 | 0.26 | -3.18 | 0.001 | 0.44 (0.27, 0.73) |
| Sufficiently active | -1.06 | 0.28 | -3.75 | <0.001 | 0.35 (0.20, 0.60) |
| Highly active | -0.67 | 0.29 | -2.30 | 0.021 | 0.51 (0.29, 0.91) |

Model 2: Including lifestyle habits (smoking history, alcohol drinking history, sitting time per day, weekly high- and mid-intensity exercise time).

CI, confidence interval; OR, odds ratio.
