# Supplementary material for: Daytime sleepiness and the association between nocturia and depressive symptoms: A cross-sectional study
Source: Medicine (Baltimore). 2026 Jul 17;105(29):e49814. doi: 10.1097/MD.0000000000049814 (PMC13384633; doi:10.1097/MD.0000000000049814)
Supplement: Supplementary file 4 [file medi-105-e49814-s004.docx]

**Table S5** Multivariable logistic regression analysis of factors associated with nocturia (Model 3).

| **Underlying diseases** | **β** | **Standard Error** | **t-value** | **P-value** | **OR (95% CI)** |
| --- | --- | --- | --- | --- | --- |
| Hypertension | 0.78 | 0.04 | 19.37 | <0.001 | 2.18 (2.02, 2.36) |
| Diabetes mellitus | 0.43 | 0.04 | 10.40 | <0.001 | 1.53 (1.41, 1.66) |
| CKD | 0.41 | 0.09 | 4.28 | <0.001 | 1.50 (1.25, 1.80) |
| Kidney stone history | 0.02 | 0.06 | 0.24 | 0.81 | 1.02 (0.90, 1.15) |

Model 3: Including underlying diseases (hypertension, diabetes mellitus, CKD, kidney stone history). CI, confidence interval; CKD, chronic kidney disease; OR, odds ratio.
