# Supplementary material for: Daytime sleepiness and the association between nocturia and depressive symptoms: A cross-sectional study
Source: Medicine (Baltimore). 2026 Jul 17;105(29):e49814. doi: 10.1097/MD.0000000000049814 (PMC13384633; doi:10.1097/MD.0000000000049814)
Supplement: Supplementary file 5 [file medi-105-e49814-s005.docx]

**Table S6** Multivariable logistic regression analysis of factors associated with nocturia (Model 4).

| **Depressive symptoms and sleep-related variables** | **β** | **Standard Error** | ***t*-value** | **P-value** | **OR (95% CI)** |
| --- | --- | --- | --- | --- | --- |
| Depression level |  |  |  |  |  |
| No depression | Reference | Reference | Reference | Reference | Reference |
| Mild depression | 0.61 | 0.05 | 12.03 | <0.001 | 1.84 (1.66, 2.03) |
| Moderate depression | 0.74 | 0.08 | 9.15 | <0.001 | 2.09 (1.79, 2.45) |
| Moderately severe depression | 0.85 | 0.13 | 6.71 | <0.001 | 2.34 (1.83, 3.01) |
| Severe depression | 0.86 | 0.19 | 4.52 | <0.001 | 2.35 (1.63, 3.42) |
| Weekday sleep hours |  |  |  |  |  |
| <6 | Reference | Reference | Reference | Reference | Reference |
| 6-9 | -0.17 | 0.06 | -2.67 | 0.008 | 0.84 (0.75, 0.96) |
| >9 | 0.44 | 0.08 | 5.54 | <0.001 | 1.56 (1.33, 1.82) |
| Daytime sleepiness score |  |  |  |  |  |
| 0 | Reference | Reference | Reference | Reference | Reference |
| 1 | -0.10 | 0.06 | -1.57 | 0.116 | 0.91 (0.81, 1.02) |
| 2 | 0.04 | 0.06 | 0.62 | 0.533 | 1.04 (0.93, 1.16) |
| 3 | 0.09 | 0.07 | 1.30 | 0.193 | 1.09 (0.96, 1.24) |
| 4 | 0.21 | 0.08 | 2.56 | 0.010 | 1.23 (1.05, 1.45) |

Model 4: Including severity of depressive symptoms, sleep duration and daytime sleepiness.

CI, confidence interval; OR, odds ratio.
