# Supplementary material for: Daytime sleepiness and the association between nocturia and depressive symptoms: A cross-sectional study
Source: Medicine (Baltimore). 2026 Jul 17;105(29):e49814. doi: 10.1097/MD.0000000000049814 (PMC13384633; doi:10.1097/MD.0000000000049814)
Supplement: Supplementary file 6 [file medi-105-e49814-s006.docx]

**Table S7** Sensitivity analysis excluding depressive symptoms, sleep duration, and daytime sleepiness from the nocturia model.

| **Variable** | **Potential mediators excluded (N=3,923)** | **Potential mediators included (N=3,923)** |
| --- | --- | --- |
| Female vs male | 1.53 (1.21, 1.94); P<0.001 | 1.34 (1.06, 1.71); P=0.016 |
| Non-Hispanic Black vs Mexican American | 1.90 (1.31, 2.76); P<0.001 | 1.95 (1.33, 2.85); P<0.001 |
| Hypertension | 1.58 (1.30, 1.92); P<0.001 | 1.46 (1.18, 1.82); P<0.001 |
| Diabetes mellitus | 1.99 (1.54, 2.58); P<0.001 | 2.00 (1.54, 2.60); P<0.001 |
| Kidney stone history | 1.03 (0.72, 1.48); P=0.858 | 0.96 (0.67, 1.38); P=0.837 |

Survey-weighted logistic regression estimates are odds ratios (95% confidence intervals). The comparison uses the same complete-case subset. The model including potential mediators is exploratory and is not interpreted as a causal adjustment model.
