# Supplementary material for: Daytime sleepiness and the association between nocturia and depressive symptoms: A cross-sectional study
Source: Medicine (Baltimore). 2026 Jul 17;105(29):e49814. doi: 10.1097/MD.0000000000049814 (PMC13384633; doi:10.1097/MD.0000000000049814)
Supplement: Supplementary file 7 [file medi-105-e49814-s007.docx]

**Table S8** Sensitivity analysis additionally adjusting for harmonized urinary incontinence variables.

| **Variable** | **Before urinary incontinence adjustment (N=3,919)** | **After urinary incontinence adjustment**  **(N=3,919)** |
| --- | --- | --- |
| Female vs male | 1.35 (1.06, 1.71); P=0.016 | 1.35 (1.06, 1.71); P=0.015 |
| Non-Hispanic Black vs Mexican American | 1.95 (1.33, 2.84); P<0.001 | 1.95 (1.33, 2.86); P<0.001 |
| Hypertension | 1.46 (1.18, 1.81); P<0.001 | 1.47 (1.18, 1.82); P<0.001 |
| Diabetes mellitus | 2.01 (1.54, 2.60); P<0.001 | 2.00 (1.53, 2.61); P<0.001 |
| Any urinary leakage | Not included | 1.06 (0.85, 1.32); P=0.585 |
| Urgency urinary incontinence | Not included | 1.04 (0.85, 1.28); P=0.691 |

Survey-weighted logistic regression estimates are odds ratios (95% confidence intervals). Any urinary leakage was defined from KIQ005, and urgency urinary incontinence was defined from KIQ044.
