# Supplementary material for: Daytime sleepiness and the association between nocturia and depressive symptoms: A cross-sectional study
Source: Medicine (Baltimore). 2026 Jul 17;105(29):e49814. doi: 10.1097/MD.0000000000049814 (PMC13384633; doi:10.1097/MD.0000000000049814)
Supplement: Supplementary file 8 [file medi-105-e49814-s008.docx]

**Table S9** Multiple linear regression analysis of nocturia frequency, daytime sleepiness, and their interaction in relation to PHQ-9 scores.

| **Variable** | **β** | **Standard Error** | ***t*-value** | **P-value** |
| --- | --- | --- | --- | --- |
| Nocturia frequency | 0.40 | 0.05 | 7.523 | <0.001 |
| Daytime sleepiness score | 1.04 | 0.04 | 23.56 | <0.001 |
| Nocturia frequency × Daytime sleepiness score | 0.12 | 0.02 | 4.84 | <0.001 |
