# Supplementary material for: Daytime sleepiness and the association between nocturia and depressive symptoms: A cross-sectional study
Source: Medicine (Baltimore). 2026 Jul 17;105(29):e49814. doi: 10.1097/MD.0000000000049814 (PMC13384633; doi:10.1097/MD.0000000000049814)
Supplement: Supplementary file 9 [file medi-105-e49814-s009.docx]

**Table S10** Direct and indirect associations among nocturia, sleep duration, and daytime sleepiness.

| **Association type** | **β** | **95% Confidence Interval** | | **P-value** | |
| --- | --- | --- | --- | --- | --- |
| Indirect association | -0.01 | (-0.01, 0.00) | <0.001 | |  |
| Direct association | 0.12 | (0.09, 0.14) | <0.001 | |  |
| Total association | 0.11 | (0.08, 0.13) | - | |  |
| Proportion accounted for | -0.05 | (-0.11, -0.03) | - | |  |
