# Supplementary material for: Daytime sleepiness and the association between nocturia and depressive symptoms: A cross-sectional study
Source: Medicine (Baltimore). 2026 Jul 17;105(29):e49814. doi: 10.1097/MD.0000000000049814 (PMC13384633; doi:10.1097/MD.0000000000049814)
Supplement: Supplementary file 10 [file medi-105-e49814-s010.docx]

**Table S12** Direct and indirect associations among nocturia, daytime sleepiness, and depressive symptoms.

| **Association type** | **β** | **95% Confidence Interval** | **P-value** |
| --- | --- | --- | --- |
| Indirect association | 0.14 | (0.09, 0.15) | <0.001 |
| Direct association | 0.51 | (0.48, 0.67) | <0.001 |
| Total association | 0.64 | (0.60, 0.80) | - |
| Proportion accounted for | 0.21 | (0.14, 0.22) | - |
