# Supplementary material for: Daytime sleepiness and the association between nocturia and depressive symptoms: A cross-sectional study
Source: Medicine (Baltimore). 2026 Jul 17;105(29):e49814. doi: 10.1097/MD.0000000000049814 (PMC13384633; doi:10.1097/MD.0000000000049814)
Supplement: Supplementary file 11 [file medi-105-e49814-s011.docx]

**Table S11** Direct and indirect associations among nocturia, sleep duration, and depressive symptoms.

| **Association type** | **β** | **95% Confidence Interval** | **P-value** |
| --- | --- | --- | --- |
| Indirect association | -0.01 | (-0.02, 0.00) | 0.083 |
| Direct association | 0.65 | (0.61, 0.80) | <0.001 |
| Total association | 0.64 | (0.60, 0.79) | - |
| Proportion accounted for | -0.01 | (-0.02, 0.00) | - |
