# Supplementary material for: Daytime sleepiness and the association between nocturia and depressive symptoms: A cross-sectional study
Source: Medicine (Baltimore). 2026 Jul 17;105(29):e49814. doi: 10.1097/MD.0000000000049814 (PMC13384633; doi:10.1097/MD.0000000000049814)
Supplement: Supplementary file 12 [file medi-105-e49814-s012.docx]

**Table S2** Demographics and clinical characteristics stratified by nocturia.

| **Variables** | **Total**  **(N=17,731)** | **Nocturia** | | | | | | **P-value** |
| --- | --- | --- | --- | --- | --- | --- | --- | --- |
|  |  | **0**  **(N=4,545)** | **1**  **(N=6,707)** | **2**  **(N=3,574)** | **3**  **(N=1,797)** | **4**  **(N=541)** | **≥5**  **(N=567)** |  |
| **Individual characteristics** |  |  |  |  |  |  |  |  |
| Age, years, mean (SD) | 51.24 (17.5) | 50.43 (18.0) | 51.52 (17.4) | 51.13 (17.1) | 51.54 (17.4) | 53.25 (16.8) | 52.38 (17.1) | 0.001 |
| Age, years, n (%) |  |  |  |  |  |  |  | <0.001 |
| 20-34 | 4,006 (22.6) | 1,144 (25.2) | 1,457 (21.7) | 783 (21.9) | 406 (22.6) | 96 (17.7) | 120 (21.2) |  |
| 35-64 | 8,980 (50.7) | 2,195 (48.3) | 3418 (51.0) | 1,875 (52.5) | 918 (51.1) | 283 (52.3) | 291 (51.3) |  |
| 65-80 | 4,745 (26.8) | 1,206 (26.5) | 1,832 (27.3) | 916 (25.6) | 473 (26.3) | 162 (29.9) | 156 (27.5) |  |
| Sex, n (%) |  |  |  |  |  |  |  | <0.001 |
| Male | 8,487 (47.9) | 2,360 (51.9) | 3,165 (47.2) | 1,649 (46.1) | 822 (45.7) | 236 (43.6) | 255 (45.0) |  |
| Female | 9,244 (52.1) | 2,185 (48.1) | 3,542 (52.8) | 1,925 (53.9) | 975 (54.3) | 305 (56.4) | 312 (55.0) |  |
| BMI, kg/m^2^, mean (SD) | 29.91 (7.4) | 27.33 (6.5) | 29.46 (7.0) | 31.86 (7.6) | 32.92 (7.6) | 33.56 (8.0) | 31.00 (7.1) | <0.001 |
| BMI, kg/m^2^, n (%) |  |  |  |  |  |  |  | <0.001 |
| <18.5 | 235 (1.3) | 100 (2.2) | 102 (1.5) | 21 (0.6) | 5 (0.3) | 2 (0.4) | 5 (0.9) |  |
| 18.5-24.9 | 4,303 (24.5) | 1,712 (37.9) | 1,654 (24.9) | 575 (16.3) | 215 (12.2) | 46 (8.7) | 101 (18.0) |  |
| 25-29.9 | 5,612 (32.0) | 1,579 (34.9) | 2,268 (34.1) | 993 (28.2) | 459 (25.9) | 138 (26.1) | 175 (31.1) |  |
| ≥30 | 7,405 (42.2) | 1,131 (25.0) | 2,628 (39.5) | 1,932 (54.9) | 1,090 (61.6) | 343 (64.8) | 281 (50.0) |  |
| Race, n (%) |  |  |  |  |  |  |  | <0.001 |
| Mexican American | 2,091 (11.8) | 572 (12.6) | 798 (11.9) | 429 (12.0) | 186 (10.4) | 52 (9.6) | 54 (9.5) |  |
| Other Hispanic | 1,910 (10.8) | 505 (11.1) | 687 (10.2) | 375 (10.5) | 207 (11.5) | 77 (14.2) | 59 (10.4) |  |
| Non-Hispanic White | 7,583 (42.8) | 1,891 (41.6) | 3,046 (45.4) | 1,496 (41.9) | 707 (39.3) | 190 (35.1) | 253 (44.6) |  |
| Non-Hispanic Black | 3,662 (20.7) | 764 (16.8) | 1,234 (18.4) | 858 (24.0) | 502 (27.9) | 161 (29.8) | 143 (25.2) |  |
| Other race | 2,485 (14.0) | 813 (17.9) | 942 (14.0) | 416 (11.6) | 195 (10.9) | 61 (11.3) | 58 (10.2) |  |
| Education, n (%) |  |  |  |  |  |  |  | <0.001 |
| Less than 9th grade | 1,236 (7.0) | 266 (5.9) | 372 (5.5) | 313 (8.8) | 184 (10.2) | 60 (11.1) | 41 (7.2) |  |
| 9-11th grade (Includes 12th grade with no diploma) | 1,755 (9.9) | 378 (8.3) | 567 (8.5) | 417 (11.7) | 217 (12.1) | 90 (16.6) | 86 (15.2) |  |
| High school graduate/GED or equivalent | 3,984 (22.5) | 866 (19.1) | 1,486 (22.2) | 872 (24.4) | 462 (25.7) | 143 (26.4) | 155 (27.4) |  |
| Some college or AA degree | 5,639 (31.8) | 1,432 (31.5) | 2,210 (33.0) | 1,141 (31.9) | 549 (30.6) | 148 (27.4) | 159 (28.1) |  |
| College graduate or above | 5,108 (28.8) | 1,602 (35.3) | 2,068 (30.9) | 829 (23.2) | 384 (21.4) | 100 (18.5) | 125 (22.1) |  |
| Marital status, n (%) |  |  |  |  |  |  |  | <0.001 |
| Married | 9,736 (54.9) | 2,512 (55.3) | 3,907 (58.3) | 1,890 (52.9) | 911 (50.7) | 247 (45.7) | 269 (47.5) |  |
| Divorced | 3,347 (18.9) | 577 (12.7) | 1,148 (17.1) | 845 (23.7) | 478 (26.6) | 153 (28.3) | 146 (25.8) |  |
| Widowed | 3,104 (17.5) | 948 (20.9) | 1,077 (16.1) | 585 (16.4) | 284 (15.8) | 92 (17.0) | 118 (20.8) |  |
| Separated | 170 (1.0) | 49 (1.1) | 52 (0.8) | 32 (0.9) | 21 (1.2) | 11 (2.0) | 5 (0.9) |  |
| Never married | 891 (5.0) | 312 (6.9) | 322 (4.8) | 143 (4.0) | 72 (4.0) | 22 (4.1) | 20 (3.5) |  |
| Living with partner | 474 (2.7) | 145 (3.2) | 198 (3.0) | 76 (2.1) | 31 (1.7) | 16 (3.0) | 8 (1.4) |  |
| Pregnancy, n (%) |  |  |  |  |  |  |  | <0.001 |
| Pregnant | 168 (4.7) | 22 (1.9) | 57 (4.0) | 41 (7.4) | 23 (9.4) | 11 (16.4) | 14 (13.3) |  |
| Not pregnant | 3,303 (92.7) | 1,113 (95.5) | 1,335 (93.5) | 501 (90.1) | 210 (86.1) | 54 (80.6) | 90 (85.7) |  |
| Unknown | 94 (2.6) | 30 (2.6) | 36 (2.5) | 14 (2.5) | 11 (4.5) | 2 (3.0) | 1 (1.0) |  |
| **Lifestyle habits** |  |  |  |  |  |  |  |  |
| Smoking history, n (%) | 3,075 (41.2) | 789 (17.4) | 1,106 (16.5) | 622 (17.4) | 335 (18.6) | 97 (17.9) | 126 (22.2) | <0.001 |
| Alcohol drinking history (drinks/week), mean (SD) | 2.59 (2.2) | 2.53 (2.1) | 2.56 (2.1) | 2.69 (2.3) | 2.71 (2.4) | 2.52 (2.3) | 2.51 (2.2) | 0.027 |
| Alcohol drinking history (drinks/week), n (%) |  |  |  |  |  |  |  | 0.012 |
| Abstinent or low-risk drinking | 6,294 (50.0) | 1,671 (49.1) | 2,550 (51.1) | 1,218 (50.9) | 550 (48.8) | 154 (49.5) | 151 (41.3) |  |
| Moderate drinking | 3,821 (30.4) | 1,034 (30.4) | 1,524 (30.5) | 709 (29.7) | 335 (29.7) | 96 (30.9) | 123 (33.6) |  |
| Heavy drinking | 2,474 (19.7) | 697 (20.5) | 918 (18.4) | 464 (19.4) | 242 (21.5) | 61 (19.6) | 92 (25.1) |  |
| Sitting time/day (min), mean (SD) | 354.39  (204.5) | 369.74  (211.3) | 351.33  (201.6) | 339.17 (197.0) | 343.82 (199.5) | 353.82 (208.3) | 398.23 (226.5) | <0.001 |
| Sitting time/day (h), n (%) |  |  |  |  |  |  |  | <0.001 |
| <3 hours/day | 2,646 (15.0) | 646 (14.3) | 998 (15.0) | 585 (16.5) | 268 (15.0) | 81 (15.1) | 68 (12.2) |  |
| 3-6 hours/day | 8,678 (49.3) | 2,095 (46.5) | 3,325 (49.8) | 1,808 (50.9) | 941 (52.7) | 264 (49.3) | 245 (43.8) |  |
| 6-9 hours/day | 3,208 (18.2) | 863 (19.1) | 1,186 (17.8) | 630 (17.7) | 303 (17.0) | 105 (19.6) | 121 (21.6) |  |
| >9 hours/day | 3,078 (17.5) | 904 (20.1) | 1,164 (17.4) | 527 (14.8) | 272 (15.2) | 86 (16.0) | 125 (22.4) |  |
| Weekly high-intensity exercise time, mean (SD) | 260.76  (348.3) | 247.85 (341.2) | 256.58  (311.1) | 266.05 (355.2) | 280.76 (417.8) | 334.11 (493.0) | 303.66 (486.4) | 0.010 |
| Weekly high-intensity exercise time, n (%) |  |  |  |  |  |  |  | 0.011 |
| Inactive | 141 (2.0) | 29 (1.4) | 52 (1.8) | 29 (2.2) | 19 (3.3) | 5 (3.6) | 7 (3.2) |  |
| Insufficiently active | 1,376 (19.1) | 387 (19.0) | 512 (17.7) | 292 (21.9) | 116 (20.0) | 18 (12.9) | 51 (23.2) |  |
| Sufficiently active | 1,813 (25.2) | 517 (25.4) | 746 (25.8) | 314 (23.5) | 147 (25.3) | 39 (27.9) | 50 (22.7) |  |
| Highly active | 3,869 (53.7) | 1,103 (54.2) | 1,576 (54.6) | 701 (52.5) | 299 (51.5) | 78 (55.7) | 112 (50.9) |  |
| Weekly mid-intensity exercise time, mean (SD) | 196.95  (284.6) | 188.66 (249.9) | 192.34  (264.3) | 207.97 (352.4) | 212.04 (299.4) | 203.64 (262.4) | 228.56 (372.6) | 0.109 |
| Weekly mid-intensity exercise time, n (%) |  |  |  |  |  |  |  | 0.278 |
| Inactive | 295 (3.9) | 71 (3.3) | 115 (3.8) | 60 (4.4) | 29 (4.6) | 8 (4.7) | 12 (6.1) |  |
| Insufficiently active | 4,065 (53.7) | 1,173 (53.9) | 1,619 (53.4) | 745 (54.4) | 332 (52.4) | 91 (53.8) | 105 (53.3) |  |
| Sufficiently active | 2,049 (27.0) | 607 (27.9) | 851 (28.1) | 340 (24.8) | 166 (26.2) | 40 (23.7) | 45 (22.8) |  |
| Highly active | 1,167 (15.4) | 326 (15.0) | 445 (14.7) | 225 (16.4) | 106 (16.7) | 30 (17.8) | 35 (17.8) |  |
| **Underlying diseases** |  |  |  |  |  |  |  |  |
| Hypertension, n (%) | 6,665 (37.6) | 1,115 (24.5) | 2,270 (33.8) | 1,680 (47.0) | 976 (54.3) | 317 (58.6) | 307 (54.1) | <0.001 |
| Diabetes mellitus, n (%) | 2,611 (14.7) | 402 (8.8) | 766 (11.4) | 671 (18.8) | 457 (25.5) | 163 (30.2) | 152 (26.8) | <0.001 |
| CKD, n (%) | 700 (4.0) | 101 (2.2) | 207 (3.1) | 184 (5.1) | 119 (6.6) | 42 (7.8) | 47 (8.3) | <0.001 |
| Kidney stone history, n (%) | 1,274 (7.2) | 310 (6.8) | 468 (7.0) | 251 (7.0) | 160 (8.9) | 41 (7.6) | 44 (7.8) | <0.001 |
| **Severity of depression** |  |  |  |  |  |  |  |  |
| Depression score, mean (SD) | 3.47 (4.4) | 2.89 (4.0) | 2.99 (4.0) | 3.74 (4.4) | 4.55 (5.0) | 5.80 (5.4) | 6.41 (5.8) | <0.001 |
| Depression level, n (%) |  |  |  |  |  |  |  | <0.001 |
| No depression | 12,833 (72.4) | 3,562 (78.4) | 5,162 (77.0) | 2,450 (68.6) | 1,124 (62.5) | 273 (50.5) | 262 (46.2) |  |
| Mild depression | 3,161 (17.8) | 650 (14.3) | 1,028 (15.3) | 751 (21.0) | 410 (22.8) | 161 (29.8) | 161 (28.4) |  |
| Moderate depression | 1,117 (6.3) | 217 (4.8) | 351 (5.2) | 252 (7.1) | 156 (8.7) | 60 (11.1) | 81 (14.3) |  |
| Moderately severe depression | 436 (2.5) | 81 (1.8) | 118 (1.8) | 84 (2.4) | 79 (4.4) | 33 (6.1) | 41 (7.2) |  |
| Severe depression | 184 (1.0) | 35 (0.8) | 48 (0.7) | 37 (1.0) | 28 (1.6) | 14 (2.6) | 22 (3.9) |  |
| **Sleep pattern and daytime sleepiness** |  |  |  |  |  |  |  |  |
| Sleep-disordered breathing symptoms, n (%) |  |  |  |  |  |  |  | 0.002 |
| Never | 8,878 (74.5) | 2,345 (76.0) | 3,410 (75.3) | 1,706 (72.3) | 892 (73.5) | 252 (70.8) | 273 (72.6) |  |
| 1-2 nights/week | 1,598 (13.4) | 405 (13.1) | 604 (13.3) | 328 (13.9) | 159 (13.1) | 46 (12.9) | 56 (14.9) |  |
| 3-4 nights/week | 776 (6.5) | 203 (6.6) | 272 (6.0) | 176 (7.5) | 75 (6.2) | 28 (7.9) | 22 (5.9) |  |
| ≥5 nights/week | 669 (5.6) | 131 (4.2) | 245 (5.4) | 151 (6.4) | 87 (7.2) | 30 (8.4) | 25 (6.6) |  |
| Weekday sleep hours, mean (SD) | 7.62 (1.6) | 7.45 (1.5) | 7.62 (1.4) | 7.71 (1.7) | 7.87 (1.8) | 7.78 (1.9) | 7.60 (2.1) | <0.001 |
| Weekday sleep hours, n (%) |  |  |  |  |  |  |  | <0.001 |
| <6 | 1,718 (9.7) | 455 (10.0) | 541 (8.1) | 382 (10.7) | 180 (10.0) | 66 (12.2) | 94 (16.6) |  |
| 6-9 | 14,048 (79.2) | 3,727 (82.0) | 5,555 (82.8) | 2,690 (75.3) | 1,326 (73.8) | 376 (69.5) | 374 (66.0) |  |
| >9 | 1,965 (11.1) | 363 (8.0) | 611 (9.1) | 502 (14.0) | 291 (16.2) | 99 (18.3) | 99(17.5) |  |
| Weekend sleep hours, mean (SD) | 8.22 (1.7) | 8.19 (1.6) | 8.24 (1.6) | 8.25 (1.9) | 8.23 (1.9) | 8.30 (1.9) | 8.00 (2.2) | 0.052 |
| Weekend sleep hours, n (%) |  |  |  |  |  |  |  | <0.001 |
| <6 | 847 (6.6) | 185 (5.9) | 255 (5.2) | 206 (7.8) | 107 (8.1) | 34 (8.9) | 60 (12.9) |  |
| 6-9 | 9,213 (71.8) | 2,350 (74.6) | 3,595 (73.8) | 1,818 (68.6) | 912 (68.9) | 240 (63.0) | 298 (64.2) |  |
| >9 | 2,778 (21.6) | 615 (19.5) | 1,018 (20.9) | 628 (23.7) | 304 (23.0) | 107 (28.1) | 106 (22.8) |  |
| Daytime sleepiness score, mean (SD) | 1.75 (1.2) | 1.63(1.2) | 1.72(1.1) | 1.82(1.2) | 1.86(1.2) | 2.10(1.2) | 2.13(1.3) | <0.001 |

Data are present in n (%) and mean (SD).

AA, associate of arts; BMI, body mass index; CKD, chronic kidney disease; GED, general educational development.
